# Sexual Minorities and Loneliness: Exploring Sexuality through Social Media and Gender–Sexuality Alliance (GSA) Supports

**DOI:** 10.3390/ijerph21030300

**Published:** 2024-03-04

**Authors:** Linda Charmaraman, Alice Zhang, Kaitlyn Wang, Becky Chen

**Affiliations:** Youth, Media, & Wellbeing Research Lab, Wellesley Centers for Women, Wellesley College, Wellesley, MA 02481, USA

**Keywords:** sexual minorities, adolescents, loneliness, social support, social media, gender–sexuality alliances (GSAs)

## Abstract

We examined online and offline social supports for sexual minority adolescents, underscoring the understudied developmental period of early adolescence and the mental outcome of loneliness. Stemming from a larger study in the northeast U.S., 967 youth participants were 26% sexual minority, 53% female, 45% male, and 2% other/nonbinary (mean age = 13.1, SD = 1.52). LGBTQ+ youth reported significantly higher levels of loneliness compared to their heterosexual counterparts. To understand potential sources of social support while exploring their sexual identities, we compared the experiences of LGBTQ+ youth at both ends of the loneliness spectrum. Gaining knowledge about their sexual orientation from LGBTQ+ organization websites, participating in gender–sexuality alliances, and using TikTok or Instagram were associated with lower levels of loneliness. Providing social support to online friends was associated with lower loneliness; however, receiving online support was not associated with lower loneliness. Furthermore, proactive social media engagement such as posting uplifting content, joining online communities, or raising awareness about social issues were associated with lower levels of loneliness. The results provide guidance on specific youth behaviors and online communities beyond a focus on screen time while highlighting the continued need for social support to ameliorate loneliness, such as gender–sexuality alliance networks.

## 1. Introduction

The U.S. Surgeon General’s recent health advisory has underscored the pervasive issue of loneliness as a critical public health concern [1]. Despite this recognition, there remains a notable gap in the understanding of how and under what circumstances marginalized groups (e.g., those who are more vulnerable to discrimination or systemic oppression), particularly LGBTQ+ individuals, experience loneliness. The impact of loneliness on LGBTQ+ individuals gained prominence during the COVID-19 pandemic, with reports indicating higher rates of loneliness and mental health challenges within this community compared to their non-LGBTQ+ counterparts [2], affecting over 60% of LGBTQ+ U.S. college students [3]. Most prior studies on loneliness have focused on older adults or college students, and research on the vulnerable adolescent transitional periods of coming out as a sexual minority is still scarce [4]. Acknowledging the dual role of technology in potentially exacerbating isolation, while also offering avenues for community-building and social support, our study focuses on exploring associations between LGBTQ+ youth loneliness and their use of online supports (e.g., social media) and offline supports (e.g., gender–sexuality alliances). By delving into this intersection, we aim to contribute nuanced insights into the ways in which LGBTQ+ youth utilize multiple sources to navigate and respond to feelings of loneliness.

### 1.1. Developing One’s Sexual Orientation Identity

Adolescent identity development is a dynamic process, characterized by fluctuating levels of exploration and commitment to an identity as one matures socially and cognitively [5]. Social media serves as a platform for experimenting with social identity, that is, a sense of who you are in relation to your group memberships. Social media is a place where adolescents articulate their evolving sense of self, seeking feedback and validation for identity experiments in order to understand their experiences as shared or unique. Social identity contributes to an individual’s self-concept, with robust group identification (e.g., sexuality) enhancing self-esteem, a sense of belonging, meaning, purpose, and efficacy in life [6]. However, strong identification with a stigmatized group (e.g., LGBTQ+) can become a source of stress. Relying on external sources for self-worth tends to reduce self-esteem and reinforce feelings of conditional acceptance [7]. These self-worth contingencies may be exacerbated positively or negatively in the social media environment, where validation is publicly displayed. It is critical to note that the impact of group affiliations on mental health is contingent upon the extent to which individuals identify strongly and internalize these affiliations as self-defining [8].

Although studies have shown that youths are coming out about their sexual orientation at earlier ages, most existing studies on sexual minority youth focus on sexual exploration with older adolescents (15–19). The average age of coming out for 13–17-year-olds is 13, and for 18–25-year-olds, the average age is 16 [9], which demonstrates that younger adolescents are now feeling more ready to come out than their slightly older counterparts. The Trevor Project [9] collected survey data from 33,993 teens showing that sexual minority youths who come out before the age of 13 experience higher rates of victimization. For fear of rejection, most youths avoid disclosing to parents until friends and associates know [10], which is a key rationale for understanding socializing contexts outside of the home for sexual minority youth.

Though there is a presumption that disclosure to parents and family members is a part of a positive sexual identity, fewer than half of ethnic-minority youth (ages 8–20) reported disclosure to family members [10]. Families of sexual minority youths are often less likely to discuss topics like sex with sexual minority youths compared to families of non-sexual minority youths, which may reflect their general discomfort or feelings of incompetency when broaching the topics [11]. For some, the inability to turn to family for guidance and understanding highlights that LGBTQ+ youth need strong social support in other aspects of their lives, including peers, mentors with shared experiences at school, or through online spaces. 

### 1.2. Social Isolation of LGBTQ+ Youth and Mental Health

Peer influence and validation are especially salient during the early adolescent developmental period as youth experience more prejudicial and homophobic behaviors [12], leading to greater difficulty in coming out as a sexual minority [13]. According to Minority Stress Theory [14], exposure to stressors such as discrimination, peer rejection, and harassment is a central cause of distress among sexual minorities. LGBTQ+ youth experience elevated rates of both in-person and online harassment, encompassing instances of homophobic and transphobic comments, and a heightened prevalence of cyberbullying when compared to their heterosexual peers [15,16]. In particular, negative responses to LGBTQ+ youth contribute to increased stress levels [16,17]. 

LGBTQ+ youth exhibit higher rates of loneliness, social isolation, and depressive symptoms than their heterosexual peers [18]. Moreover, LGBTQ+ youth grappling with loneliness are less likely to reach out for help regarding their mental health concerns [19]. According to the Trevor Project’s 2023 National Survey on LGBTQ+ Youth Mental Health [20], 56% of LGBTQ+ youth aged 13–24 in the U.S. (n = 35,208) expressed a desire for mental health care but faced barriers in obtaining care. The primary reported obstacle was the fear of discussing mental health concerns with others. 

Sexual minority and gender minority youths demonstrate lower suicide attempt rates when exposed to supportive environments [20]. While youth identify online sites as the most commonly accessible environment supportive of LGBTQ+ identity (68% of sexual minority and 70% of gender minority youth), schools were the next frequently reported site (54% of sexual minority and 52% of gender minority youth), suggesting the importance of organizations within schools specifically devoted to LGBTQ+ awareness and advocacy [20]. As a counterpoint to Minority Stress Theory, a minority strengths-based approach examines how strengths-based variables (such as feeling pride in one’s LGBTQ+ community) is interrelated with mental and physical health outcomes [21]. A prior study of over 17,000 youth conducted by the Human Rights Campaign [22] demonstrated that higher levels of social support are associated with higher levels of LGBTQ+ pride, and lack of support decreases pride in one’s sexual identity and increases the risk of depression. While studies that link LGBTQ+ pride with depression rates are emerging, little is known about how identifying strongly with one’s sexual orientation and/or having LGBTQ+ “pride” is associated with loneliness. Further research is imperative to identify specific offline and online social supports that can foster affirming environments that may accentuate pride. 

### 1.3. Social Supports for Identity Development of Young Sexual Minority Youth

A conceptual review highlights two key avenues through which LGBTQ+ youth build connection: online engagement and participation in gender–sexuality alliances (GSAs) [19]. Online platforms serve as particularly valuable resources for youth facing discrimination and social isolation in their in-person lives by providing opportunities to explore sexual minority experiences and mental health in a safer environment [19]. In comparison to non-LGBTQ+ youth, LGBTQ+ youth are also significantly more likely to develop close online friendships, with 50% reporting such connections compared to 19% of their non-LGBTQ+ counterparts [23]. Through GSAs, LGBTQ+ youth can cultivate connection and a greater sense of agency by developing supportive social relationships and engaging in advocacy initiatives [19]. GSAs may thus play a pivotal role in bolstering support for sexual and gender minority students who currently receive limited social support, thereby fortifying these student-led organizations as sites of affirmation and belonging [19]. Although studies have demonstrated how often LGBTQ+ youth make connections via online friendships and supportive GSAs, little is known about associations between these social supports and their mental health outcomes, such as loneliness.

#### 1.3.1. Online Peer Support via Social Media 

Sexual minority youth are avid users of social media, which can be partly attributed to the disproportionate risks and limited access to social support they face in other social contexts, such as at home, in school, and in the community [24]. Social media can play a unique role as a place for LGBTQ+ youth to disclose their identities to their curated audiences, which can sometimes exclude family members [25]. While family is an integral component of LGBTQ+ youth’s experiences of disclosing and understanding their identities, existing research pays less attention to the role of peers. A study of over 5000 teens revealed that sexual minority adolescents believed that their online friends were more supportive than their in-person friends [26]. Sexual minorities have demonstrated resiliency when using social media to explore their sexual identity, which can support their mental health [27]. In a cross-sectional survey study of early adolescents, sexual minority youth had higher rates of loneliness and had a preference to use social media to maintain web-based communities, particularly those that are small and close-knit in nature, to reduce loneliness [18]. To ensure that they avoid negative interactions and the pressure to conform to heteronormative expectations that they may face offline, LGBTQ+ youth use de-identifying names and locations and change privacy measures on their social media [28], especially when considering the possibility of negative reactions from family members online. From a systematic review of 18 studies that focused on social media use and LGBTQ youth well-being, Instagram, Tumblr, and Twitter were listed as the commonly mentioned platforms that facilitated identity expression and exploration due to their higher identity disclosure features. Many LGBTQ+ youth aged 16+ reported preferring Tumblr and Twitter to Facebook to specifically express their LGBTQ identity because of Facebook’s restrictive policies and audiences [28]. In recent years, younger youth have gravitated toward sites other than Facebook (e.g., Instagram, TikTok, YouTube), suggesting a need to re-examine current social media platform trends amongst LGBTQ+ early adolescents.

#### 1.3.2. Gender–Sexuality Alliances

Gender–sexuality alliances (GSAs) are extracurricular school organizations, largely present in the U.S., that aim to provide safe spaces for participants to discuss LGBTQ+ experiences and participate in LGBTQ+ advocacy [29]. As such, while GSAs are typically student-led groups and operate differently across schools, they each strive to serve as a means of social support for LGBTQ+ students whose experiences and needs may not otherwise be recognized. These organizations provide opportunities for community engagement, education, and leadership development for LGBTQ+ youth and allies and can cover a wide range of topics, including advocating for greater awareness of different LGBTQ+ identities, providing support to those facing discrimination or seeking to come out, and co-learning about health subjects [29,30]. GLSEN’s 2019 National School Climate Survey found that schools with GSAs were associated with lower levels of LGBTQ+ youth victimization than schools without GSAs, and LGBTQ+ youth reported feeling safer and more accepted by their peers [31]. Additionally, GSA advisors can provide valuable social support, guidance, and mentorship that influences students’ well-being: one survey study indicates that participants (ages 14–19) reported increased levels of hope after GSA meetings in which their advisors appeared more responsive to their needs and goals [32]. This underscores the importance of designated spaces for students to explore LGBTQ+ identity alongside classmates and advisors, potentially reducing feelings of loneliness. Greater involvement in advocacy and access to GSA resources in high school are associated with more frequent engagement in conversations surrounding sexual health, mental health, and substance use [33], especially for those who experienced greater levels of victimization. Additionally, adolescents who engaged in advocacy and received emotional support and resources from GSAs reported increased levels of hope near the end of the school year compared to the start of the school year—this was particularly true for students who had faced victimization, highlighting the potential benefits of GSAs for LGBTQ+ youth who are at greater risk of loneliness [29]. 

One longitudinal study of 14–16-year-olds reported that perceived support from a GSA helped buffer against the relationship between homophobic cyberbullying and mental health effects [34]. Furthermore, involvement in a GSA is associated with increased civic engagement, raising awareness, and advocating for LGBTQ+ experiences. For both LGBTQ+ youth and allies, GSAs can help counter myths surrounding sexual health through education, school initiatives, and advocacy on a broader scale [35]. For instance, a qualitative study of Canadian high school allies described how students observed a lack of LGBTQ+ experiences in school curricula, including sexual health topics, and sought to fill those gaps [35]. Another study of students (grades 8–12) reported that they helped organize LGBTQ+ advocacy events in school or in the community (e.g., leading workshops, honoring days such as Transgender Day of Remembrance), or worked to make a difference in their community [33]. A greater sense of agency was also found to help mediate the relationship between GSA participation and general civic engagement [33]. 

### 1.4. LGBTQ+ Youth and Online Community Engagement

According to the longitudinal Adolescent Brain Cognitive Development (ABCD) study, while youth in general (10–14 years) spend approximately 7.19 h per day online on average, sexual minority and questioning youth reported spending 1.5–4 additional hours online every day compared to their heterosexual peers [36]. Sexual minority youth also spend a significantly greater amount of time on their cell phones than heterosexual youth every day, including being on social media for nearly half an hour longer daily [36]. The well-established greater online presence may reflect LGBTQ+ youth’s ability to find support and resources online that are absent in their in-person lives [4]. For instance, Gillig [37] found that LGBTQ+ youth (ages 13–21) used virtual camp space to socialize, find connections, and build their support networks where their sexual identity is celebrated and safe, which allowed youths to cope during the COVID-19 crisis. A qualitative study of 25 transgender youth (ages 15–18) in the midwestern U.S. discussed how using social media reduced feelings of isolation and contributed to users feeling validation and hope [38]. Participants also described how anonymity provides a chance for transgender youth to be open about their experiences. LGBTQ+ youth tend to gravitate toward platforms such as Instagram, Tumblr, and Twitter because they provide opportunities to connect with other LGBTQ+ peers while remaining anonymous and being able to use pseudonyms. One study conducted through surveys (n = 1304) and interviews (n = 23) with LGBTQ+ Australian youth, found that the majority of LGBTQ+ youth on Tumblr used the site to connect with strangers and bond over shared fandoms or topics of interest [39]. Only 3% of participants reported using Tumblr to connect with existing friends, in contrast to 87% of participants using Snapchat and 83% on Facebook, a site that requires disclosing more of one’s identity [28,39]. While using social media, LGBTQ+ youth also curate how they present themselves. A recent systematic review looking at identity management by LGBTQ+ youth details how they have reported using multiple accounts, being selective about their social media audiences, and using online platforms to receive and provide social support [28]. Additional research on other specific sites, such as TikTok and Instagram, is essential for a deeper understanding of why LGBTQ+ youth gravitate toward certain sites and how those sites play a role in reducing loneliness. 

### 1.5. Online Community and Civic Engagement

A national cross-sectional survey study in the U.S. working with youth 14–18 years of age found that most LGBTQ+ youth in the sample (93.7%) engage in a form of LGBTQ+ civic engagement online, from sharing information to organizing initiatives about LGBTQ+ rights [40]. LGBTQ+ youth are approximately twice as likely to participate in online civic engagement than non-LGBTQ+ youth; GLSEN reports that 77% of LGBTQ+ youth (n = 5680, 13–18 years old) reported being a member of an online community that advocates for social issues [23]. 22% reported participating in civic engagement only online, emphasizing the importance of online spaces for activism. The likelihood of LGBTQ+ youth participating in civic engagement online compared to offline is greater for several potential reasons [41]. LGBTQ+ adolescents and young adults (n = 6309; 14–29 years) across the U.S. and Canada who participated in the online survey Project #Queery reported engaging with the LGBTQ+ community more online than in-person; they felt that it was significantly safer to participate in community engagement online and were more likely to access and engage with LGBTQ+ resources available online, including resources about physical and mental health [41]. Exposure to such resources and validating online communities can help youth feel empowered to speak about LGBTQ+ issues online, providing support to other LGBTQ+ individuals while affirming their own identity and well-being [42]. Many LGBTQ+ youth also describe vocalizing personal experiences and advocating for change online, engaging in a form of “everyday activism” while not necessarily identifying as “activists” themselves. Since the dominant literature on online civic engagement focuses on youth aged 13+, studies are scarce when it comes to even younger LGBTQ+ adolescents.

### 1.6. Current Study

Early adolescent LGBTQ+ youth aged 10–15 remain an understudied group, as most social media research focuses primarily on older adolescents and young adults [4,38]. For instance, in a recent systematic review [28], only one study out of 18 included younger teens—and none of them included tweens (aged 10-12). Younger adolescents may be considered a particularly vulnerable group due to the recency of acquiring their first smartphone and the lack of opportunity to develop social media literacy, self-regulation skills, and social competence that older users may have gained over time [43]. Additionally, LGBTQ+ youth who are coming out earlier than 13 are at higher risk of suicide [9]. We seek to extend prior findings by focusing on the understudied developmental period of adolescence, starting from age 10, and the mental outcome of loneliness, examining associations with specific social media behaviors (not just screen time) within sexual minority youth. We explore both online and offline sources of social and sexual identity support these youth receive and any relationship to loneliness.

### 1.7. Research Questions

Comparing heteronormative with LGBTQ+ youth:

1. Are LGBTQ+ youth significantly lonelier?

Within LGBTQ+ subsample:

2. In what ways are less lonely LGBTQ+ youth significantly different from more lonely LGBTQ+ counterparts, such as (a) exploring sexual orientation (e.g., the strength of identifying with sexual orientation, what sources of social support and education do they receive about their sexual orientation) and (b) online community support (e.g., social media platforms used, social media help-seeking preferences, giving and receiving online social support, and online civic engagement)?

## 2. Materials and Methods

### 2.1. Procedures

The current study was part of a larger ongoing longitudinal survey in the northeast U.S. about social technology and adolescent health [18,44]. For the current cross-sectional study, out of 40 schools that were invited to join the study, nine schools were interested in joining the pilot. Three diverse school sites participated in this current study were originally recruited because their diverse public school composition (e.g., racially, ethnically, culturally, linguistically, and socioeconomically) reflects the demographics of their communities. After obtaining institutional and school district IRB approvals, online surveys were completed during an open advisory or wellness period (or completed at home) in the fall of 2020. A parent study disclosure and opt-out consent form were distributed ahead of time by the principal, wellness coordinator, or PTO representative. Students provided assent on the first page of the survey. Partially due to social distancing during the COVID-19 pandemic, students were attending schools in a hybrid formation (i.e., online some days, in-person on other days). Students who were absent on the days of in-person data collection were invited to take the survey at home at their convenience. School staff proctored the survey, while study staff were available to answer questions from staff or students through a Zoom or Google Meet link. Several $25 raffle prizes were distributed to students for participating, and schools received a $600 honorarium for access to their students.

### 2.2. Measures

#### 2.2.1. Sexual Orientation

We classified sexual orientation as a binary variable: ‘0’ denoted heterosexual and ‘1’ represented sexual minority. Participants who reported attraction to the opposite sex were coded as ‘0’ (heterosexual), whereas those attracted to both sexes, the same sex, neither sex or who identified as unsure/questioning or other were coded as ‘1’ (sexual minority).

#### 2.2.2. Loneliness

To assess feelings of loneliness, we utilized a three-item scale adapted from the UCLA Loneliness Scale [45]. This instrument gauges perceptions of social isolation through statements prompting participants to indicate the frequency of specific feelings: ‘How often do you feel…’ that you lack companionship, left out, and isolated from others. 

Responses were recorded using a 3-point Likert scale measuring frequency: ‘1’ denoted ‘Hardly ever’, ‘2’ signified ‘Some of the time’, and ‘3’ represented ‘Often’ (Cronbach α = 0.81). To compare higher and lower levels of loneliness, we dichotomized participants based on the group’s average loneliness response (mean = 1.77). Scores greater than the group average were categorized as ‘1’ (higher loneliness), while those below the average were classified as ‘0’ (lower loneliness).

#### 2.2.3. Strength of Sexual Identity

Participants were asked to reflect on the primary facets of their self-identity. Those who indicated that their sexual orientation was a significant aspect of their identity were coded as ‘1’. In contrast, those who did not view it as a central component were coded as ‘0’.

#### 2.2.4. Sources of Sexual Orientation and Gender Identity Education

We inquired about the online or social media resources participants used to learn about their sexual orientation or gender identity. The options included “None”, “LGBTQ+ organization websites”, “GSA clubs”, “YouTube”, “TikTok”, “Reddit”, “Tumblr”, “Twitter”, or “Instagram”. Participants could select multiple sources as applicable. We dichotomized each item: ‘1’ represented ‘Yes, I have used this source’ and ‘0’ denoted ‘No, I have not used this source’.

#### 2.2.5. Social Media Help-Seeking Preferences

Participants were asked, “If you could help design a new app or online game that could assist teens with their social media use, which one(s) would you be most interested in how…” to make more friends online, to avoid mean comments and users, to provide social support to others, to reduce feelings of loneliness or depression, to reduce time spent on the internet or phones, to improve self-esteem, to take more breaks from social media, teens can help make the world a better place to live, and to cope during COVID-19 social distancing. Participant responses were captured using a 3-point Likert scale measuring interest level: ‘1’ denoted ‘Not interested’, ‘2’ represented ‘Somewhat interested’, and ‘3’ signified ‘Very interested’.

#### 2.2.6. Positive Social Media Engagement

To understand how adolescents engage with social media positively, we asked, “How frequently do you use the internet or social media to…” assist with your homework or a class project, post content aimed at uplifting others, react positively when friends share good news, offer support to friends when they share distressing or sad news, organize an event for your friends or community, join a group or online community that made you feel less isolated, and raise awareness about a social issue you are passionate about. Participant responses were captured using a 6-point Likert scale measuring frequency: ‘1’ corresponded to ‘Never/Does not apply to me’, ‘2’ to ‘Every few days’, ‘3’ to ‘Once a day’, ‘4’ to ‘Every few hours’, ‘5’ to ‘Every hour’, and ‘6’ to ‘More than every hour’.

#### 2.2.7. Giving and Receiving Emotional Support on Social Media

To assess the exchange of emotional support among friends on social media, participants were presented with two specific questions: “How often do you receive emotional support from your friends on social media?” and “On social media, how often have you tried to help a friend who needed emotional support, for instance, by posting something to cheer them up (e.g., ‘Take care of yourself’)?” Participants provided their responses using a 4-point scale: ‘1’ indicated ‘Never’, ‘2’ represented ‘Rarely’, ‘3’ was ‘Sometimes’, and ‘4’ denoted ‘All the time’.

#### 2.2.8. Gender

Participants were requested to self-identify their gender using a numeric scale: ‘1’ denoted ‘Male’, ‘2’ denoted ‘Female’, and ‘3’ denoted ‘Other’. Individuals who selected ‘Other’ were provided with the option to specify their identified gender in a write-in format. A total of 15 out of 967 participants chose ‘Other’, and due to the small subgroup size, these individuals were excluded from the final statistical analyses.

### 2.3. Sample Description

Our study encompasses 967 students (967/1271, 76.08% total response rate), aged between 10 and 17 years (average age 13.09, SD 1.52 years), sourced from three schools from the Northeastern U.S. (see Table 1 for demographics). The urban and suburban cities represented in this sample are characterized as 52% college-educated, one-third to two-thirds Democratic, 48–53% Catholic, 20–32% foreign-born, with an 8–11% child poverty rate. From the respondents who completed the survey with the analytical variables, 837 individuals with known sexual orientation were categorized into one of two groups based on self-reported sexual orientation: heterosexual (584/837, 69.69%) or sexual minority (253/837, 30.19%). For the purpose of this study, the term ‘sexual minority’ includes individuals who reported attraction to the same sex, both sexes, neither sex, those who are unsure or questioning, or those who specified other types of sexual attraction.

**Figure 1:**
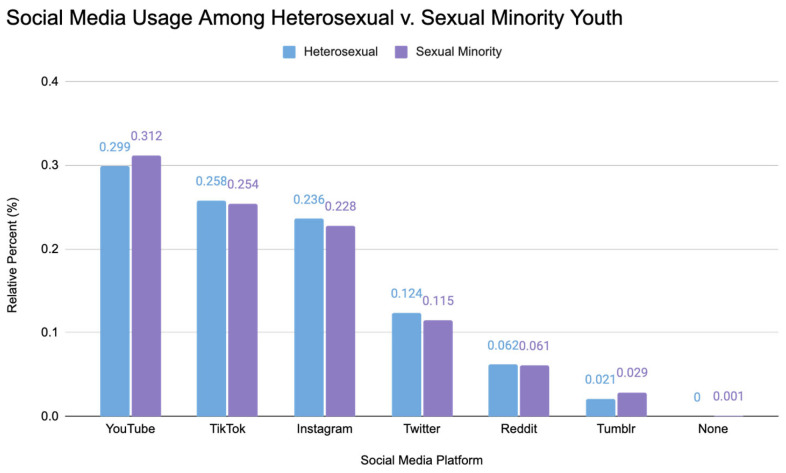
Social media usage among heterosexual v. sexual minority youth. This bar chart illustrates the relative percentages of heterosexual and sexual minority youth engaging with various social media platforms. The percentages are calculated based on the total number of individuals for their respective group for each platform.

Sexual minority participants used similar social media sites to heterosexual participants, as shown below in Figure 1.

### 2.4. Analysis Plan

In the data analysis phase of our study, we employed binary logistic regression models within the R Statistical Software (v. 4.2.2) [46] to explore the interrelationships among sexual minority identity, sources of knowledge regarding sexual orientation, and online behavior. We used linear regression to investigate research question #1, the relationship between levels of loneliness and sexual orientation. To examine research question #2, we created a binary response variable, dichotomized as higher versus lower loneliness to understand nuances within a subgroup that had higher levels of loneliness than average. Missing values were handled through a list-wise deletion approach, ensuring the inclusion of only complete cases in the analysis. To account for potential confounding factors and reduce the risk of omitted variable bias, all models controlled for the effects of age and gender.

#### Covariates

**Figure 2:**
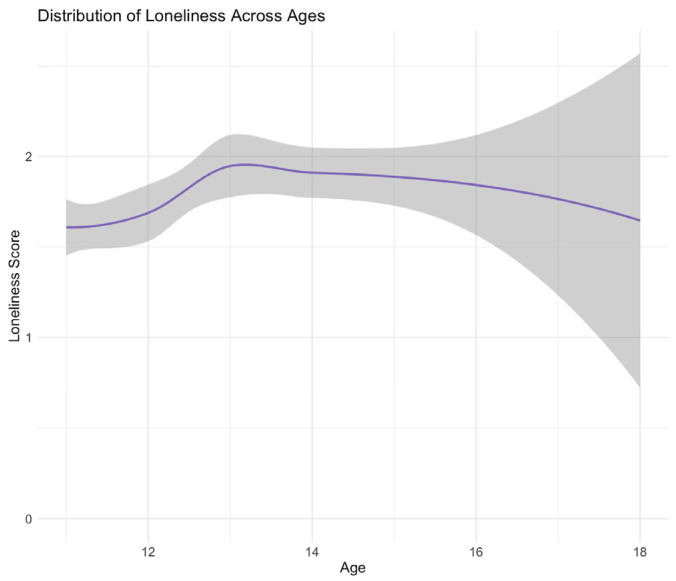
Distribution of loneliness scores across age for sexual minority youth. This line graph depicts the distribution of loneliness scores across a spectrum of ages for sexual minority youth. The shading in gray highlights variations within each age group. Notably, an increase in age, particularly between the ages of 16 and 18, is associated with a discernible spike in the variability of loneliness scores.

**Figure 3:**
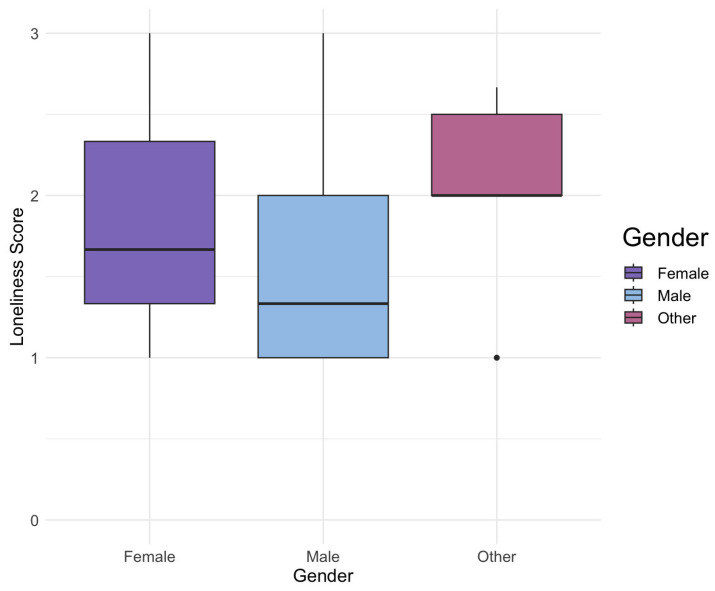
Distribution of loneliness scores across gender categories for sexual minority youth. This graphic presents three boxplots illustrating the distribution of loneliness scores across different gender categories—female, male, and other. The visual representation underscores noticeable distinctions: the other category exhibits less variability, with a concentration in higher loneliness scores, while both male and female categories display greater variability. Additionally, the mean loneliness score for females surpasses that of males.

Age was treated as a continuous quantitative variable, representing each participant’s self-reported age, including month, day, and year (see Figure 2). Gender, a categorical variable (see Figure 3), was derived from participant responses indicating ‘female’, ‘male’, or ‘other’. Subsequently, we recoded this information into a binary variable, distinguishing ‘female’ from ‘male’. However, due to the limited representation of the ‘other’ category (6/253, 2.37%) within our sexual minority sample and (15/967, 1.55%) in our overall population, the insufficient sample size prompted us to exclude these cases from our analysis.

## 3. Results

### 3.1. Are LGBTQ+ Youth More Lonely?

**Figure 4:**
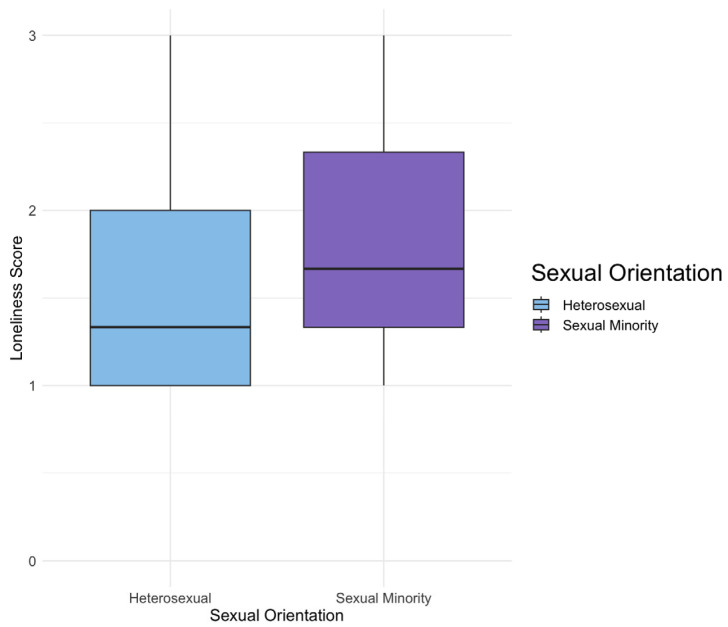
These box plots illustrate the distribution of loneliness scores for the entire survey sample, categorized by sexual orientation. The data reveals a diverse range of loneliness scores among both sexual minority and heterosexual youth. Notably, the average loneliness score for sexual minority youth is higher in comparison to their heterosexual counterparts.

Sexual minorities, on average, reported significantly higher levels of loneliness compared to their heterosexual counterparts (β = 0.224, 95% CI 0.134–0.314, *p* < 0.001). See Figure 4 below.

### 3.2. Sexual Orientation Exploration and Education

We found several factors associated with the range of loneliness levels among LGBTQ+ youth. Specifically, having a strong identification with their sexual orientation identity had a protective effect (β = −0.158, OR = 0.853, 95% CI 0.734–0.992, *p* = <0.001). Participating in GSAs was associated with lower loneliness levels (β = −0.336, OR = 0.715, 95% CI 0.545–0.937, *p* = 0.0165). Gaining knowledge about their sexual orientation from LGBTQ+ organization websites (β = −0.219, OR= 0.804, 95% CI 0.677–0.953, *p* = 0.0128), using TikTok (β = −0.292, OR = 0.747, 95% CI 0.653–0.854, *p* < 0.001), or using Instagram (β = −0.252, OR = 0.777, 95% CU 0.657–0.920, *p* = 0.00369) were associated with lower levels of loneliness. Conversely, having a specific source of information about their sexual orientation was associated with lower levels of loneliness (β = 0.289, OR = 1.335, 95% CI 1.174–1.519, *p* > 0.001) compared to having no source to turn to at all. Other popular platforms such as YouTube, Reddit, Tumblr, and Twitter showed no significant association with loneliness levels among this group.

### 3.3. Support and Engagement within Online Communities

Sexual minority youth who provided online support to friends experienced less loneliness (β = −0.104, OR = 0.901, 95% CI 0.840–0.967, *p* = 0.00418). However, receiving such support was not found to be significantly associated with loneliness levels.

Less lonely sexual minority youth expressed a preference for the following type of social media app guidance, including friendship-building (β = −0.107, OR = 0.899, 95% CI 0.818–0.987, *p* = 0.0267), providing social support (β = −0.106, OR = 0.899, 95% CI 0.826–0.979, *p* = 0.0149), addressing feelings of loneliness or depression (β = −0.146, OR = 0.865, 95% CI 0.800–0.935, *p* < 0.001), and bolstering self-esteem (β = −0.125, OR = 0.883, 95% CI 0.817–0.954, *p* = 0.00176). Furthermore, proactive engagement with social media platforms, such as posting uplifting content, “feel-good” posts (β = −0.0568, OR = 0.945, 95% CI 0.900–0.992, *p* = 0.0225), joining groups or online communities that reduce feelings of isolation (β = −0.0511, OR = 0.883, 95% CI 0.817–0.954, *p* = 0.00369), or online civic engagement, such as raising awareness about social issues they care about (β = −0.0786, OR = 0.924, 95% CI 0.883, 0.968, *p* < 0.001), were also associated with lower levels of loneliness among sexual minority youth.

## 4. Discussion

Consistent with existing literature on the higher prevalence of loneliness among LGBTQ+ adolescents [4,19], the current study found that LGBTQ+ youth face significantly higher levels of loneliness compared to their heterosexual peers, at an age younger than previously found in the literature that is typically focused on older adolescents. When comparing LGBTQ+ youth with their heteronormative peers in terms of levels of loneliness at face value, we found that this aligned with the Minority Stress Theory. Notably, within the LGBTQ+ youth cohort, our findings also revealed a strengths-based nuance—those who identified more strongly with their LGBTQ+ identity exhibited lower levels of loneliness. This trend implies a protective effect: individuals who consider their LGBTQ+ identity as integral to their sense of self experience lower levels of loneliness relative to their LGBTQ+ peers with lower sexual identity pride levels. Drawing from a more minority strengths-based framework [21] that was previously found to be protective of depressive symptoms [22], internalizing LGBTQ+ identity as an important part of their identity was a protective factor in the mental outcome of loneliness.

When it comes to LGBTQ+ adolescents’ experiences in the offline world, active participation in GSAs was associated with reduced levels of loneliness. While GSAs vary widely as student-led organizations, their common goal of providing spaces for members to build community, learn about LGBTQ+ topics, and advocate for issues that matter to them helps cultivate empowering environments that may be absent elsewhere in their lives. Participating in GSAs has been shown to have a protective effect against online victimization and correlates with higher self-esteem and a greater sense of belonging at school [34,47]. In a recent longitudinal evaluation of the impact of GSAs, GSA members expressed more hope following meetings in which they felt more supported by their advisors and the organization overall, and after participating in more leadership roles [32]. Thus, it is important for members to feel that they have the chance to get involved with GSA leadership, strive to meet the needs of members, and have access to responsive advisors. The majority of GSA advisors report having had little to no professional training regarding LGBTQ+ topics, and specific training in preventing loneliness should be made more accessible to support the well-being of LGBTQ+ youth [32]. Additionally, as we examine an understudied population of early adolescents, our findings support the need for greater support for GSA participation starting in middle school. While GSAs are more common in high schools than middle schools, middle school students attend GSA meetings more often than high schoolers, which might be linked to the phenomenon of LGBTQ+ middle schoolers experiencing more discrimination while in school [31,47]. Our findings underscore the urgent need for widespread support of GSAs in educational settings, particularly in middle schools. By providing accessible training for advisors and fostering an inclusive environment, we can actively contribute to the creation of supportive communities that significantly alleviate loneliness and promote the holistic development of LGBTQ+ youth.

For LGBTQ+ youth, particularly those who face stigma in in-person relationships, building community through online spaces is critical. Learning about their identities from LGBTQ+ organization websites and on social media was associated in our findings with less loneliness. Corroborating research that demonstrates the correlation between online social connection and decreased sense of isolation in LGBTQ+ youth [38], we identified how youth engage in specific online behaviors that are linked to lower levels of loneliness, including the desire to use social media to build friendships, seeking out resources on addressing loneliness, and developing greater self-esteem. Additionally, we found that helping others online was associated with lower levels of loneliness, but this was not true for receiving support. This may point to the prevalence of LGBTQ+ youth community engagement and a sense of empowerment when supporting others [42]. LGBTQ+ youth may find greater fulfillment and agency when providing online support compared to when they are receiving it. Prior neurobiological research has demonstrated stress-relieving and reward-activating benefits of providing social support to others [48]. A possible alternative view is that receiving support may be associated with other confounding mental health factors, such as depressive symptoms with prior research noting a decrease in self-esteem when receiving help from others [49]. More research regarding the benefits and challenges of giving and receiving support online in a vulnerable subgroup such as LGBTQ+ youth is needed.

Examining specific sites, we found that TikTok and Instagram were associated with lower levels of loneliness, whereas no association was found for YouTube, Reddit, Tumblr, and Twitter. This contrasts with a recent systematic review [28] that Instagram, Tumblr, and Twitter were the social media sites that LGBTQ+ youth most gravitated to in order to explore LGBTQ content, particularly for anonymity purposes. Since the studies that comprised the review were published between 2012 and 2021, the shift may represent a societal shift in online safe community spaces and affordances of the platforms themselves. TikTok was the most downloaded app in early 2020, which is the year that the current data was collected. Potential reasons for the gravitation toward these sites could include the high number of LGBTQ+ influencers on TikTok and the practice of acquiring multiple accounts on Instagram to compartmentalize their social networks. This trend may align with a recent Trevor Project report in which LGBTQ+ youth identified on which sites they felt most affirmed in their identities [50]. TikTok (53% for youth of color; 45% for white youth) and Instagram (42% for youth of color; 38% for white youth) were the two sites on which respondents reported feeling safest and most validated. Additional research on why that is the case, and consideration of how racial-ethnic identity intersects with LGBTQ+ identity, is needed. Furthermore, studies that distinguish between the online preferences of sexual minorities on specific social media sites are necessary for a deeper understanding of why youth are drawn to particular platforms, and how those platforms can better support their wellbeing and identities.

### 4.1. Limitations

These results must be taken in the context of being possibly unique to the earlier COVID-19 pandemic period in the fall of 2020. While our findings provide a cross-sectional overview of LGBTQ+ online engagement and participation in GSAs and their relation to loneliness with youth in the northeast region of the U.S., longitudinal studies in other regions within and beyond the U.S. on such topics are needed for further understanding of any bidirectional influences between social media use and the mental health of LGBTQ+ youth. Existing studies on loneliness among LGBTQ+ youth are predominantly conducted within the U.S. [19]. While GSAs do exist outside of Anglo-Saxon countries, there are few research studies examining their impact on students, or they follow university-age students outside the scope of our focus. Additionally, limited scholarship on this topic globally may reflect varying social and legal norms, such as the criminalization of being LGBTQ+ in a number of countries [51,52].

A key objective of our study is to pinpoint factors that potentially mitigate feelings of loneliness among these adolescents. By creating a binary variable for loneliness within the LGBTQ+ sample, our approach aimed to differentiate between those who experience more intense loneliness from those who feel it less acutely. Our assessment did not ask about youth’s experiences participating in GSAs beyond whether they have spoken with GSA members about their sexual identities. Further research is needed to understand whether less lonely sexual minorities join GSAs or if the GSAs themselves help reduce social isolation and loneliness.

It is important to note that while this study focuses primarily on sexual minority youths, gender minority youths experience gender-specific stresses based on their gender nonconformity rather than their sexual orientation [53].

### 4.2. Future Directions

From our sample, we found that those who experienced their first sexual encounter at a younger age were significantly more likely to feel lonely. This correlation between loneliness and the age of first sexual experience underscores that youth need to have access to sexual health resources, especially considering the widespread absence or opposition to comprehensive sex education in school curricula that include LGBTQ+ sexual health. In addition to teachers, LGBTQ+ youth should receive greater support from professionals in other avenues of life, including healthcare settings. Pediatricians and other clinicians have expressed a need for training on how to better serve LGBTQ+ youth, so such patients feel seen and receive necessary clinical care [54,55]. More research about early sexual debut and loneliness within LGBTQ+ youth is needed. Future research might explore why receiving social support is not as protective as providing social support in LGBTQ+ youths, which may be a critical intervenable dimension to understand. Analysis of the content experienced on socially supportive social media sites for LGBTQ+ youth may raise awareness about the specific social and emotional preferences for safe online environments. Future studies examining loneliness and social media use in LGBTQ+ comparing early versus later adolescence would be critical to understand unique developmental processes within a period of time often treated as homogenous. Because prior research had demonstrated that gender minority youth experience higher rates of loneliness [56], more comprehensive studies examining different categories of sexual orientations and gender minorities and how they use online and offline supports is warranted.

## 5. Conclusions

LGBTQ+ youth spend a significantly greater number of hours on their phones than heterosexual youth, and using a strengths-based perspective, the online world can provide access to community and validation that may not exist in their day-to-day in-person lives. The current study reveals a nuanced connection between LGBTQ+ youths’ online and offline engagement and emotional well-being, suggesting that digital interactions on such sites as TikTok and Instagram as well as active involvement in gender–sexuality alliances might serve as a buffer against feelings of seclusion. Whether participating in-person or virtually, socially supportive communities that enhance LGBTQ+ adolescents’ positive identification with their sexual orientation may be key to reducing social isolation in this vulnerable subgroup where loneliness is heightened. LGBTQ+ youths’ preference for social media to improve their self-esteem, find friends, provide social support to their friends, and combat loneliness and depression also provide motivations behind specific social media behaviors among less lonely LGBTQ+ youth that may underpin their relatively lower loneliness levels.

## Figures and Tables

**Table 1:** Participant demographic data (N = 967).

Characteristic	Participants
Age (years)	
Value, range	10–17
Value, mean (SD)	13.09 (1.52)
Gender n (%)	
Female	515 (53.26)
Male	437 (45.19)
Other	15 (1.55)
Sexual minority (yes; n = 837 with known sexual orientation), n (%)	253 (26.16)
Not sure or questioning	119 (47.04)
Attracted to both sexes	75 (29.64)
Not attracted to either sex	13 (5.14)
Attracted to the same sex	22 (8.7)
Other	24 (9.49)
Grade n (%)	
Sixth	239 (24.72)
Seventh	204 (21.1)
Eighth	224 (23.16)
Ninth	155 (16.03)
Tenth	144 (14.89)
Missing Information	1 (0.1)
Race or ethnicity n (%)	
White	499 (51.6)
Black	81 (8.38)
Hispanic	157 (16.24)
Asian	55 (5.69)
Native American	41 (4.24)
Biracial	63 (6.51)
Middle Eastern	30 (3.1)
Other	38 (3.93)
Missing Information	3 (0.31)
Free or reduced-price lunch eligibility, n (%)	386 (39.92)

## Data Availability

The data presented in this study are available on request from the corresponding author.
